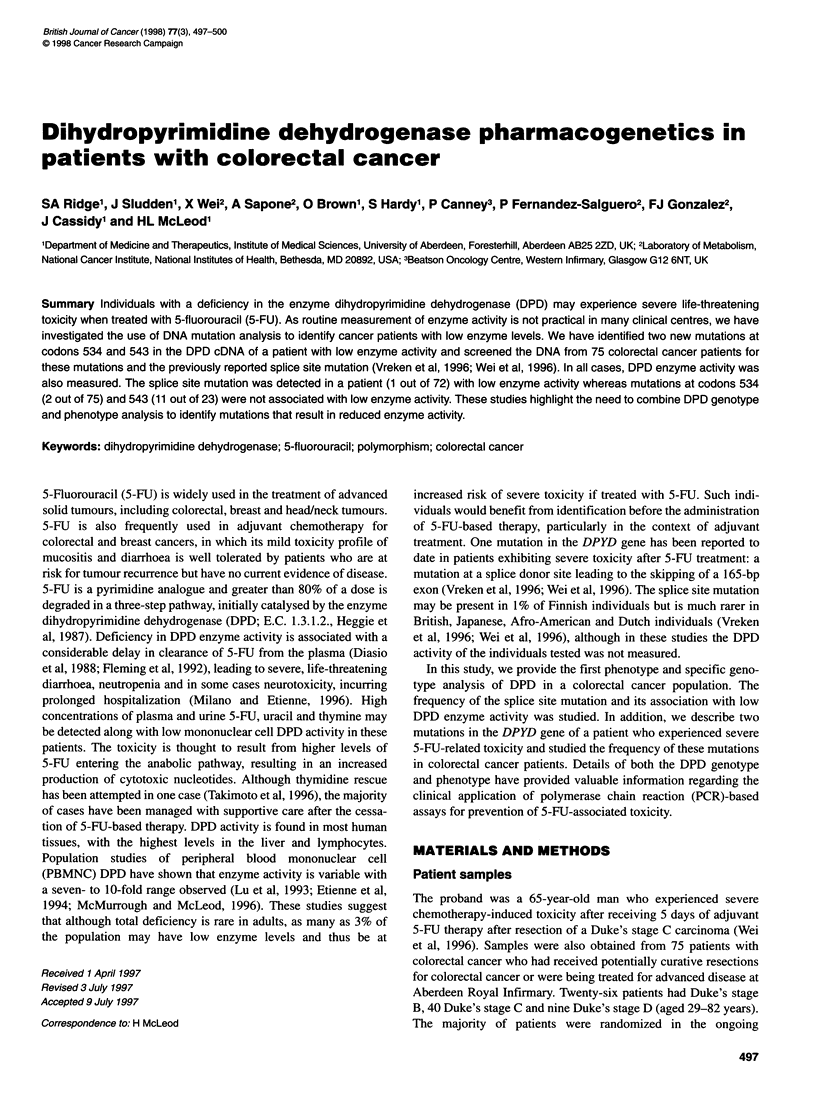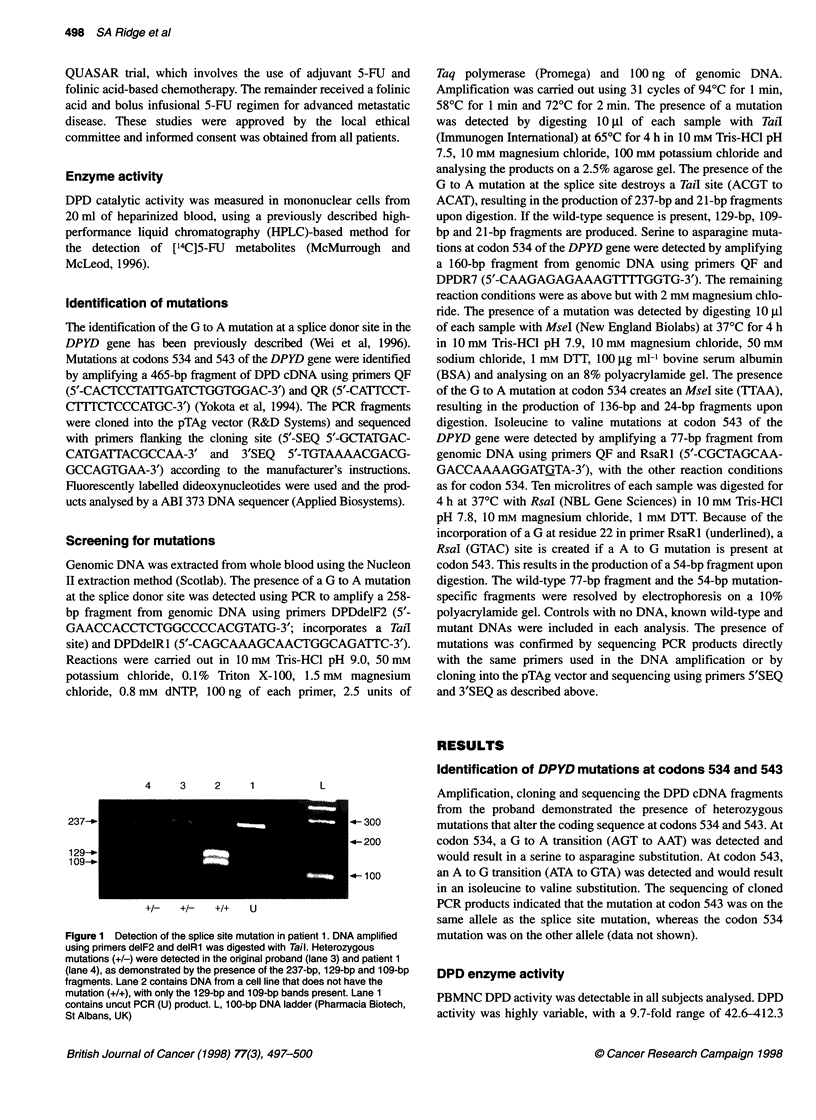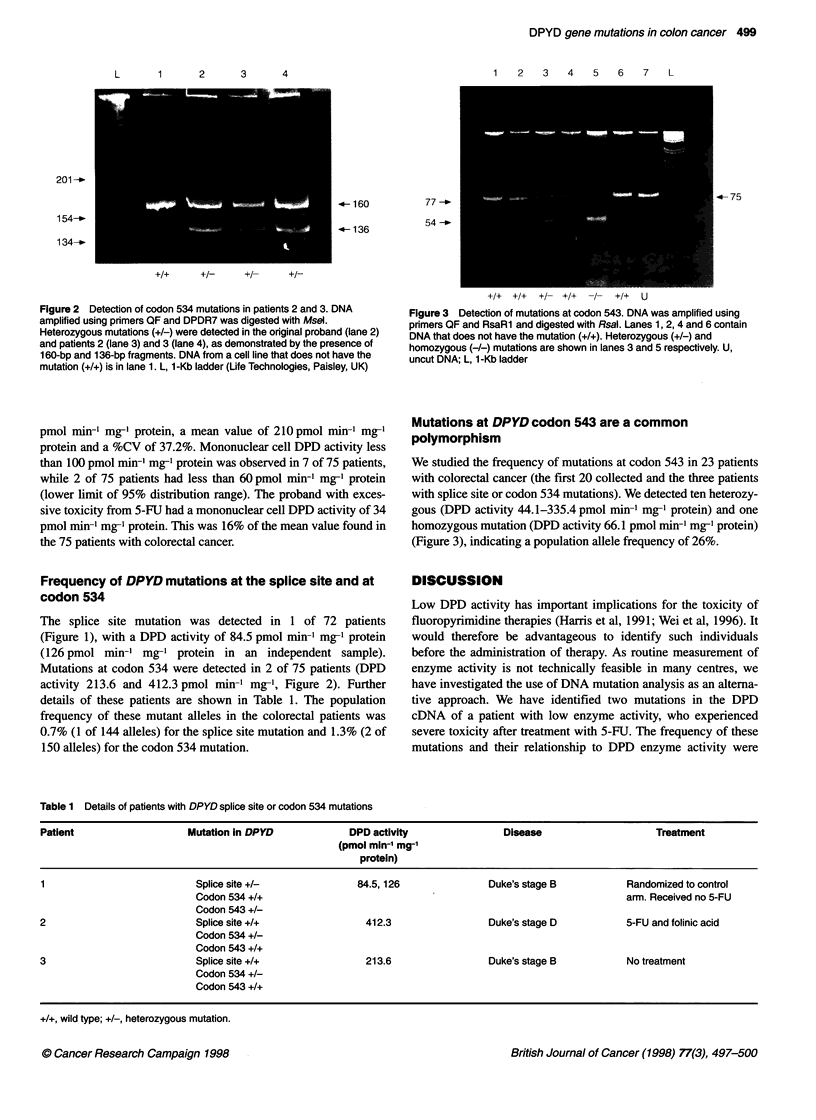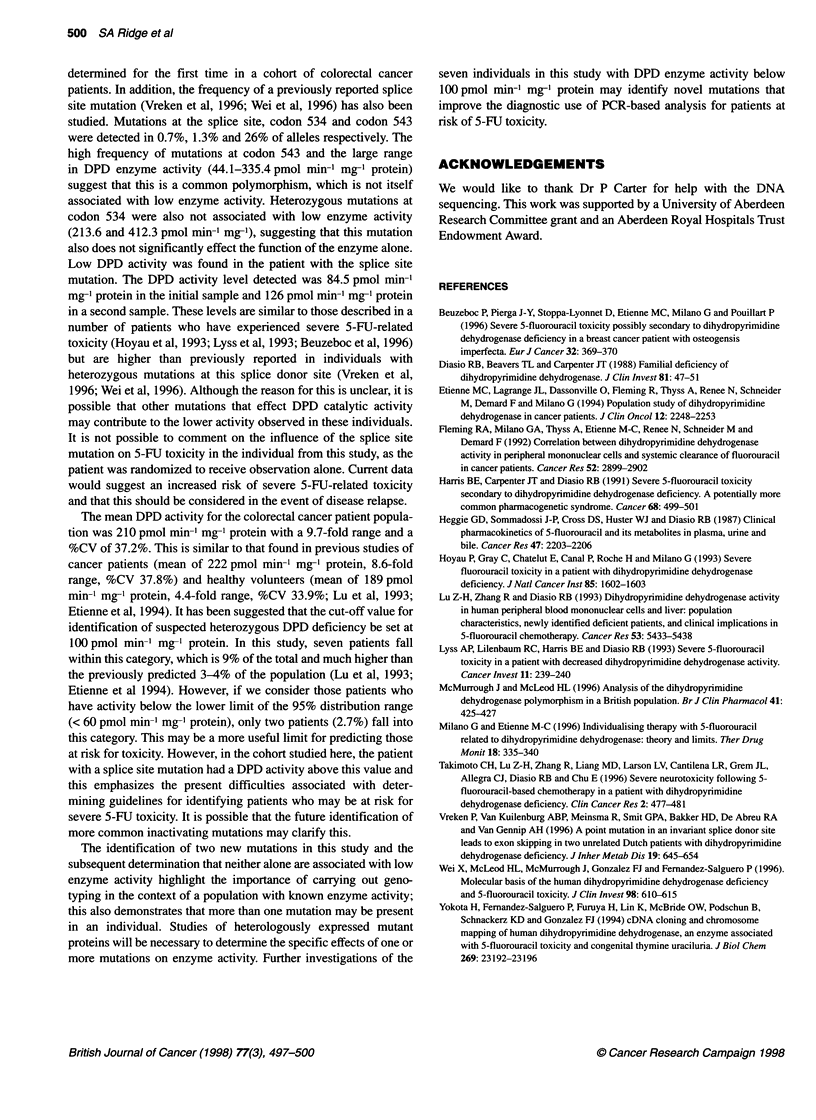# Dihydropyrimidine dehydrogenase pharmacogenetics in patients with colorectal cancer.

**DOI:** 10.1038/bjc.1998.79

**Published:** 1998

**Authors:** S. A. Ridge, J. Sludden, X. Wei, A. Sapone, O. Brown, S. Hardy, P. Canney, P. Fernandez-Salguero, F. J. Gonzalez, J. Cassidy, H. L. McLeod

**Affiliations:** Department of Medicine and Therapeutics, Institute of Medical Sciences, University of Aberdeen, Foresterhill, UK.

## Abstract

**Images:**


British Journal of Cancer (1998) 77(3), 497–500
© 1998 Cancer Research Campaign

### Dihydropyrimidine dehydrogenase pharmacogenetics in patients with colorectal cancer

**SA Ridge[1], J Sludden[1], X Wei[2], A Sapone[2], O Brown[1], S Hardy[1], P Canney[3], P Fernandez-Salguero[2], FJ Gonzalez[2], J Cassidy[1] and HL McLeod[1]**

[1]Department of Medicine and Therapeutics, Institute of Medical Sciences, University of Aberdeen, Foresterhill, Aberdeen AB25 2ZD, UK; [2]Laboratory of Metabolism, National Cancer Institute, National Institutes of Health, Bethesda, MD 20892, USA; [3]Beatson Oncology Centre, Western Infirmary, Glasgow G12 6NT, UK

**Summary** Individuals with a deficiency in the enzyme dihydropyrimidine dehydrogenase (DPD) may experience severe life-threatening toxicity when treated with 5-fluorouracil (5-FU). As routine measurement of enzyme activity is not practical in many clinical centres, we have investigated the use of DNA mutation analysis to identify cancer patients with low enzyme levels. We have identified two new mutations at codons 534 and 543 in the DPD cDNA of a patient with low enzyme activity and screened the DNA from 75 colorectal cancer patients for these mutations and the previously reported splice site mutation (Vreken et al, 1996; Wei et al, 1996). In all cases, DPD enzyme activity was also measured. The splice site mutation was detected in a patient (1 out of 72) with low enzyme activity whereas mutations at codons 534 (2 out of 75) and 543 (11 out of 23) were not associated with low enzyme activity. These studies highlight the need to combine DPD genotype and phenotype analysis to identify mutations that result in reduced enzyme activity.

**Keywords:** dihydropyrimidine dehydrogenase; 5-fluorouracil; polymorphism; colorectal cancer

5-Fluorouracil (5-FU) is widely used in the treatment of advanced solid tumours, including colorectal, breast and head/neck tumours. 5-FU is also frequently used in adjuvant chemotherapy for colorectal and breast cancers, in which its mild toxicity profile of mucositis and diarrhoea is well tolerated by patients who are at risk for tumour recurrence but have no current evidence of disease. 5-FU is a pyrimidine analogue and greater than 80% of a dose is degraded in a three-step pathway, initially catalysed by the enzyme dihydropyrimidine dehydrogenase (DPD; E.C. 1.3.1.2., Heggie et al, 1987). Deficiency in DPD enzyme activity is associated with a considerable delay in clearance of 5-FU from the plasma (Diasio et al, 1988; Fleming et al, 1992), leading to severe, life-threatening diarrhoea, neutropenia and in some cases neurotoxicity, incurring prolonged hospitalization (Milano and Etienne, 1996). High concentrations of plasma and urine 5-FU, uracil and thymine may be detected along with low mononuclear cell DPD activity in these patients. The toxicity is thought to result from higher levels of 5-FU entering the anabolic pathway, resulting in an increased production of cytotoxic nucleotides. Although thymidine rescue has been attempted in one case (Takimoto et al, 1996), the majority of cases have been managed with supportive care after the cessation of 5-FU-based therapy. DPD activity is found in most human tissues, with the highest levels in the liver and lymphocytes. Population studies of peripheral blood mononuclear cell (PBMNC) DPD have shown that enzyme activity is variable with a seven- to 10-fold range observed (Lu et al, 1993; Etienne et al, 1994; McMurrough and McLeod, 1996). These studies suggest that although total deficiency is rare in adults, as many as 3% of the population may have low enzyme levels and thus be at increased risk of severe toxicity if treated with 5-FU. Such individuals would benefit from identification before the administration of 5-FU-based therapy, particularly in the context of adjuvant treatment. One mutation in the *DPYD* gene has been reported to date in patients exhibiting severe toxicity after 5-FU treatment: a mutation at a splice donor site leading to the skipping of a 165-bp exon (Vreken et al, 1996; Wei et al, 1996). The splice site mutation may be present in 1% of Finnish individuals but is much rarer in British, Japanese, Afro-American and Dutch individuals (Vreken et al, 1996; Wei et al, 1996), although in these studies the DPD activity of the individuals tested was not measured.

*Received 1 April 1997*
*Revised 3 July 1997*
*Accepted 9 July 1997*

*Correspondence to:* H McLeod

In this study, we provide the first phenotype and specific genotype analysis of DPD in a colorectal cancer population. The frequency of the splice site mutation and its association with low DPD enzyme activity was studied. In addition, we describe two mutations in the *DPYD* gene of a patient who experienced severe 5-FU-related toxicity and studied the frequency of these mutations in colorectal cancer patients. Details of both the DPD genotype and phenotype have provided valuable information regarding the clinical application of polymerase chain reaction (PCR)-based assays for prevention of 5-FU-associated toxicity.

### MATERIALS AND METHODS

### Patient samples

The proband was a 65-year-old man who experienced severe chemotherapy-induced toxicity after receiving 5 days of adjuvant 5-FU therapy after resection of a Duke's stage C carcinoma (Wei et al, 1996). Samples were also obtained from 75 patients with colorectal cancer who had received potentially curative resections for colorectal cancer or were being treated for advanced disease at Aberdeen Royal Infirmary. Twenty-six patients had Duke's stage B, 40 Duke's stage C and nine Duke's stage D (aged 29–82 years). The majority of patients were randomized in the ongoing

QUASAR trial, which involves the use of adjuvant 5-FU and folinic acid-based chemotherapy. The remainder received a folinic acid and bolus infusional 5-FU regimen for advanced metastatic disease. These studies were approved by the local ethical committee and informed consent was obtained from all patients.

### Enzyme activity

DPD catalytic activity was measured in mononuclear cells from 20 ml of heparinized blood, using a previously described high-performance liquid chromatography (HPLC)-based method for the detection of [$^{14}$C]5-FU metabolites (McMurrough and McLeod, 1996).

### Identification of mutations

The identification of the G to A mutation at a splice donor site in the *DPYD* gene has been previously described (Wei et al, 1996). Mutations at codons 534 and 543 of the *DPYD* gene were identified by amplifying a 465-bp fragment of DPD cDNA using primers QF (5′-CACTCCTATTGATCTGGTGGAC-3′) and QR (5′-CATTCCT-CTTTCTCCCATGC-3′) (Yokota et al, 1994). The PCR fragments were cloned into the pTAg vector (R&D Systems) and sequenced with primers flanking the cloning site (5′-SEQ 5′-GCTATGAC-CATGATTACGCCAA-3′ and 3′SEQ 5′-TGTAAAACGACG-GCCAGTGAA-3′) according to the manufacturer's instructions. Fluorescently labelled dideoxynucleotides were used and the products analysed by a ABI 373 DNA sequencer (Applied Biosystems).

### Screening for mutations

Genomic DNA was extracted from whole blood using the Nucleon II extraction method (Scotlab). The presence of a G to A mutation at the splice donor site was detected using PCR to amplify a 258-bp fragment from genomic DNA using primers DPDdelF2 (5′-GAACCACCTCTGGCCCCACGTATG-3′; incorporates a *Tai*I site) and DPDdelR1 (5′-CAGCAAAGCAACTGGCAGATTC-3′). Reactions were carried out in 10 mM Tris-HCl pH 9.0, 50 mM potassium chloride, 0.1% Triton X-100, 1.5 mM magnesium chloride, 0.8 mM dNTP, 100 ng of each primer, 2.5 units of *Taq* polymerase (Promega) and 100 ng of genomic DNA. Amplification was carried out using 31 cycles of 94°C for 1 min, 58°C for 1 min and 72°C for 2 min. The presence of a mutation was detected by digesting 10 µl of each sample with *Tai*I (Immunogen International) at 65°C for 4 h in 10 mM Tris-HCl pH 7.5, 10 mM magnesium chloride, 100 mM potassium chloride and analysing the products on a 2.5% agarose gel. The presence of the G to A mutation at the splice site destroys a *Tai*I site (ACGT to ACAT), resulting in the production of 237-bp and 21-bp fragments upon digestion. If the wild-type sequence is present, 129-bp, 109-bp and 21-bp fragments are produced. Serine to asparagine mutations at codon 534 of the *DPYD* gene were detected by amplifying a 160-bp fragment from genomic DNA using primers QF and DPDR7 (5′-CAAGAGAGAAAGTTTTGGTG-3′). The remaining reaction conditions were as above but with 2 mM magnesium chloride. The presence of a mutation was detected by digesting 10 µl of each sample with *Mse*I (New England Biolabs) at 37°C for 4 h in 10 mM Tris-HCl pH 7.9, 10 mM magnesium chloride, 50 mM sodium chloride, 1 mM DTT, 100 µg ml$^{-1}$ bovine serum albumin (BSA) and analysing on an 8% polyacrylamide gel. The presence of the G to A mutation at codon 534 creates an *Mse*I site (TTAA), resulting in the production of 136-bp and 24-bp fragments upon digestion. Isoleucine to valine mutations at codon 543 of the *DPYD* gene were detected by amplifying a 77-bp fragment from genomic DNA using primers QF and RsaR1 (5′-CGCTAGCAA-GACCAAAAGGATGTA-3′), with the other reaction conditions as for codon 534. Ten microlitres of each sample was digested for 4 h at 37°C with *Rsa*I (NBL Gene Sciences) in 10 mM Tris-HCl pH 7.8, 10 mM magnesium chloride, 1 mM DTT. Because of the incorporation of a G at residue 22 in primer RsaR1 (underlined), a *Rsa*I (GTAC) site is created if a A to G mutation is present at codon 543. This results in the production of a 54-bp fragment upon digestion. The wild-type 77-bp fragment and the 54-bp mutation-specific fragments were resolved by electrophoresis on a 10% polyacrylamide gel. Controls with no DNA, known wild-type and mutant DNAs were included in each analysis. The presence of mutations was confirmed by sequencing PCR products directly with the same primers used in the DNA amplification or by cloning into the pTAg vector and sequencing using primers 5′SEQ and 3′SEQ as described above.

**Figure 1** Detection of the splice site mutation in patient 1. DNA amplified using primers delF2 and delR1 was digested with *Tai*I. Heterozygous mutations (+/−) were detected in the original proband (lane 3) and patient 1 (lane 4), as demonstrated by the presence of the 237-bp, 129-bp and 109-bp fragments. Lane 2 contains DNA from a cell line that does not have the mutation (+/+), with only the 129-bp and 109-bp bands present. Lane 1 contains uncut PCR (U) product. L, 100-bp DNA ladder (Pharmacia Biotech, St Albans, UK)

### RESULTS

### Identification of *DPYD* mutations at codons 534 and 543

Amplification, cloning and sequencing the DPD cDNA fragments from the proband demonstrated the presence of heterozygous mutations that alter the coding sequence at codons 534 and 543. At codon 534, a G to A transition (AGT to AAT) was detected and would result in a serine to asparagine substitution. At codon 543, an A to G transition (ATA to GTA) was detected and would result in an isoleucine to valine substitution. The sequencing of cloned PCR products indicated that the mutation at codon 543 was on the same allele as the splice site mutation, whereas the codon 534 mutation was on the other allele (data not shown).

### DPD enzyme activity

PBMNC DPD activity was detectable in all subjects analysed. DPD activity was highly variable, with a 9.7-fold range of 42.6–412.3

© Cancer Research Campaign 1998

**Figure 2** Detection of codon 534 mutations in patients 2 and 3. DNA amplified using primers QF and DPDR7 was digested with *MseI*. Heterozygous mutations (+/–) were detected in the original proband (lane 2) and patients 2 (lane 3) and 3 (lane 4), as demonstrated by the presence of 160-bp and 136-bp fragments. DNA from a cell line that does not have the mutation (+/+) is in lane 1. L, 1-Kb ladder (Life Technologies, Paisley, UK)

**Figure 3** Detection of mutations at codon 543. DNA was amplified using primers QF and RsaR1 and digested with *RsaI*. Lanes 1, 2, 4 and 6 contain DNA that does not have the mutation (+/+). Heterozygous (+/–) and homozygous (–/–) mutations are shown in lanes 3 and 5 respectively. U, uncut DNA; L, 1-Kb ladder pmol $min^{-1}$ $mg^{-1}$ protein, a mean value of 210 pmol $min^{-1}$ $mg^{-1}$ protein and a %CV of 37.2%. Mononuclear cell DPD activity less than 100 pmol $min^{-1}$ $mg^{-1}$ protein was observed in 7 of 75 patients, while 2 of 75 patients had less than 60 pmol $min^{-1}$ $mg^{-1}$ protein (lower limit of 95% distribution range). The proband with excessive toxicity from 5-FU had a mononuclear cell DPD activity of 34 pmol $min^{-1}$ $mg^{-1}$ protein. This was 16% of the mean value found in the 75 patients with colorectal cancer.

### Frequency of *DPYD* mutations at the splice site and at codon 534

The splice site mutation was detected in 1 of 72 patients (Figure 1), with a DPD activity of 84.5 pmol $min^{-1}$ $mg^{-1}$ protein (126 pmol $min^{-1}$ $mg^{-1}$ protein in an independent sample). Mutations at codon 534 were detected in 2 of 75 patients (DPD activity 213.6 and 412.3 pmol $min^{-1}$ $mg^{-1}$, Figure 2). Further details of these patients are shown in Table 1. The population frequency of these mutant alleles in the colorectal patients was 0.7% (1 of 144 alleles) for the splice site mutation and 1.3% (2 of 150 alleles) for the codon 534 mutation.

### Mutations at *DPYD* codon 543 are a common polymorphism

We studied the frequency of mutations at codon 543 in 23 patients with colorectal cancer (the first 20 collected and the three patients with splice site or codon 534 mutations). We detected ten heterozygous (DPD activity 44.1–335.4 pmol $min^{-1}$ $mg^{-1}$ protein) and one homozygous mutation (DPD activity 66.1 pmol $min^{-1}$ $mg^{-1}$ protein) (Figure 3), indicating a population allele frequency of 26%.

### DISCUSSION

Low DPD activity has important implications for the toxicity of fluoropyrimidine therapies (Harris et al, 1991; Wei et al, 1996). It would therefore be advantageous to identify such individuals before the administration of therapy. As routine measurement of enzyme activity is not technically feasible in many centres, we have investigated the use of DNA mutation analysis as an alternative approach. We have identified two mutations in the DPD cDNA of a patient with low enzyme activity, who experienced severe toxicity after treatment with 5-FU. The frequency of these mutations and their relationship to DPD enzyme activity were

**Table 1** Details of patients with *DPYD* splice site or codon 534 mutations

| Patient | Mutation in *DPYD* | DPD activity (pmol $min^{-1}$ $mg^{-1}$ protein) | Disease | Treatment |
|---|---|---|---|---|
| 1 | Splice site +/–<br>Codon 534 +/+<br>Codon 543 +/– | 84.5, 126 | Duke's stage B | Randomized to control arm. Received no 5-FU |
| 2 | Splice site +/+<br>Codon 534 +/–<br>Codon 543 +/+ | 412.3 | Duke's stage D | 5-FU and folinic acid |
| 3 | Splice site +/+<br>Codon 534 +/–<br>Codon 543 +/+ | 213.6 | Duke's stage B | No treatment |

+/+, wild type; +/–, heterozygous mutation.

determined for the first time in a cohort of colorectal cancer patients. In addition, the frequency of a previously reported splice site mutation (Vreken et al, 1996; Wei et al, 1996) has also been studied. Mutations at the splice site, codon 534 and codon 543 were detected in 0.7%, 1.3% and 26% of alleles respectively. The high frequency of mutations at codon 543 and the large range in DPD enzyme activity (44.1–335.4 pmol $min^{-1}$ $mg^{-1}$ protein) suggest that this is a common polymorphism, which is not itself associated with low enzyme activity. Heterozygous mutations at codon 534 were also not associated with low enzyme activity (213.6 and 412.3 pmol $min^{-1}$ $mg^{-1}$), suggesting that this mutation also does not significantly effect the function of the enzyme alone. Low DPD activity was found in the patient with the splice site mutation. The DPD activity level detected was 84.5 pmol $min^{-1}$ $mg^{-1}$ protein in the initial sample and 126 pmol $min^{-1}$ $mg^{-1}$ protein in a second sample. These levels are similar to those described in a number of patients who have experienced severe 5-FU-related toxicity (Hoyau et al, 1993; Lyss et al, 1993; Beuzeboc et al, 1996) but are higher than previously reported in individuals with heterozygous mutations at this splice donor site (Vreken et al, 1996; Wei et al, 1996). Although the reason for this is unclear, it is possible that other mutations that effect DPD catalytic activity may contribute to the lower activity observed in these individuals. It is not possible to comment on the influence of the splice site mutation on 5-FU toxicity in the individual from this study, as the patient was randomized to receive observation alone. Current data would suggest an increased risk of severe 5-FU-related toxicity and that this should be considered in the event of disease relapse.

The mean DPD activity for the colorectal cancer patient population was 210 pmol $min^{-1}$ $mg^{-1}$ protein with a 9.7-fold range and a %CV of 37.2%. This is similar to that found in previous studies of cancer patients (mean of 222 pmol $min^{-1}$ $mg^{-1}$ protein, 8.6-fold range, %CV 37.8%) and healthy volunteers (mean of 189 pmol $min^{-1}$ $mg^{-1}$ protein, 4.4-fold range, %CV 33.9%; Lu et al, 1993; Etienne et al, 1994). It has been suggested that the cut-off value for identification of suspected heterozygous DPD deficiency be set at 100 pmol $min^{-1}$ $mg^{-1}$ protein. In this study, seven patients fall within this category, which is 9% of the total and much higher than the previously predicted 3–4% of the population (Lu et al, 1993; Etienne et al 1994). However, if we consider those patients who have activity below the lower limit of the 95% distribution range (< 60 pmol $min^{-1}$ $mg^{-1}$ protein), only two patients (2.7%) fall into this category. This may be a more useful limit for predicting those at risk for toxicity. However, in the cohort studied here, the patient with a splice site mutation had a DPD activity above this value and this emphasizes the present difficulties associated with determining guidelines for identifying patients who may be at risk for severe 5-FU toxicity. It is possible that the future identification of more common inactivating mutations may clarify this.

The identification of two new mutations in this study and the subsequent determination that neither alone are associated with low enzyme activity highlight the importance of carrying out genotyping in the context of a population with known enzyme activity; this also demonstrates that more than one mutation may be present in an individual. Studies of heterologously expressed mutant proteins will be necessary to determine the specific effects of one or more mutations on enzyme activity. Further investigations of the seven individuals in this study with DPD enzyme activity below 100 pmol $min^{-1}$ $mg^{-1}$ protein may identify novel mutations that improve the diagnostic use of PCR-based analysis for patients at risk of 5-FU toxicity.

### ACKNOWLEDGEMENTS

We would like to thank Dr P Carter for help with the DNA sequencing. This work was supported by a University of Aberdeen Research Committee grant and an Aberdeen Royal Hospitals Trust Endowment Award.

### REFERENCES

Beuzeboc P, Pierga J-Y, Stoppa-Lyonnet D, Etienne MC, Milano G and Pouillart P (1996) Severe 5-fluorouracil toxicity possibly secondary to dihydropyrimidine dehydrogenase deficiency in a breast cancer patient with osteogensis imperfecta. *Eur J Cancer* **32**: 369–370

Diasio RB, Beavers TL and Carpenter JT (1988) Familial deficiency of dihydropyrimidine dehydrogenase. *J Clin Invest* **81**: 47–51

Etienne MC, Lagrange JL, Dassonville O, Fleming R, Thyss A, Renee N, Schneider M, Demard F and Milano G (1994) Population study of dihydropyrimidine dehydrogenase in cancer patients. *J Clin Oncol* **12**: 2248–2253

Fleming RA, Milano GA, Thyss A, Etienne M-C, Renee N, Schneider M and Demard F (1992) Correlation between dihydropyrimidine dehydrogenase activity in peripheral mononuclear cells and systemic clearance of fluorouracil in cancer patients. *Cancer Res* **52**: 2899–2902

Harris BE, Carpenter JT and Diasio RB (1991) Severe 5-fluorouracil toxicity secondary to dihydropyrimidine dehydrogenase deficiency. A potentially more common pharmacogenetic syndrome. *Cancer* **68**: 499–501

Heggie GD, Sommadossi J-P, Cross DS, Huster WJ and Diasio RB (1987) Clinical pharmacokinetics of 5-fluorouracil and its metabolites in plasma, urine and bile. *Cancer Res* **47**: 2203–2206

Hoyau P, Gray C, Chatelut E, Canal P, Roche H and Milano G (1993) Severe fluorouracil toxicity in a patient with dihydropyrimidine dehydrogenase deficiency. *J Natl Cancer Inst* **85**: 1602–1603

Lu Z-H, Zhang R and Diasio RB (1993) Dihydropyrimidine dehydrogenase activity in human peripheral blood mononuclear cells and liver: population characteristics, newly identified deficient patients, and clinical implications in 5-fluorouracil chemotherapy. *Cancer Res* **53**: 5433–5438

Lyss AP, Lilenbaum RC, Harris BE and Diasio RB (1993) Severe 5-fluorouracil toxicity in a patient with decreased dihydropyrimidine dehydrogenase activity. *Cancer Invest* **11**: 239–240

McMurrough J and McLeod HL (1996) Analysis of the dihydropyrimidine dehydrogenase polymorphism in a British population. *Br J Clin Pharmacol* **41**: 425–427

Milano G and Etienne M-C (1996) Individualising therapy with 5-fluorouracil related to dihydropyrimidine dehydrogenase: theory and limits. *Ther Drug Monit* **18**: 335–340

Takimoto CH, Lu Z-H, Zhang R, Liang MD, Larson LV, Cantilena LR, Grem JL, Allegra CJ, Diasio RB and Chu E (1996) Severe neurotoxicity following 5-fluorouracil-based chemotherapy in a patient with dihydropyrimidine dehydrogenase deficiency. *Clin Cancer Res* **2**: 477–481

Vreken P, Van Kuilenburg ABP, Meinsma R, Smit GPA, Bakker HD, De Abreu RA and Van Gennip AH (1996) A point mutation in an invariant splice donor site leads to exon skipping in two unrelated Dutch patients with dihydropyrimidine dehydrogenase deficiency. *J Inher Metab Dis* **19**: 645–654

Wei X, McLeod HL, McMurrough J, Gonzalez FJ and Fernandez-Salguero P (1996). Molecular basis of the human dihydropyrimidine dehydrogenase deficiency and 5-fluorouracil toxicity. *J Clin Invest* **98**: 610–615

Yokota H, Fernandez-Salguero P, Furuya H, Lin K, McBride OW, Podschun B, Schnackerz KD and Gonzalez FJ (1994) cDNA cloning and chromosome mapping of human dihydropyrimidine dehydrogenase, an enzyme associated with 5-fluorouracil toxicity and congenital thymine uraciluria. *J Biol Chem* **269**: 23192–23196

© Cancer Research Campaign 1998